# Advanced Glycation End Products-Induced Alzheimer’s Disease and Its Novel Therapeutic Approaches: A Comprehensive Review

**DOI:** 10.7759/cureus.61373

**Published:** 2024-05-30

**Authors:** Dhivya Kothandan, Daniel S Singh, Goutham Yerrakula, Backkiyashree D, Pratibha N, Vincy Santhana Sophia B, Ramya A, Sapthami Ramya VG, Keshavini S, Jagadheeshwari M

**Affiliations:** 1 Department of Pharmacy Practice, C.L. Baid Metha College of Pharmacy, Chennai, IND; 2 School of Pharmacy, Faculty of Health Sciences, JSS Academy of Higher Education and Research, Vacoas, MUS

**Keywords:** rage, oxidative stress, ages, alzheimer’s disease, dementia

## Abstract

Advanced glycation end products (AGEs) accumulate in the brain, leading to neurodegenerative conditions such as Alzheimer's disease (AD). The pathophysiology of AD is influenced by receptors for AGEs and toll-like receptor 4 (TLR4). Protein glycation results in irreversible AGEs through a complicated series of reactions involving the formation of Schiff's base, the Amadori reaction, followed by the Maillard reaction, which causes abnormal brain glucose metabolism, oxidative stress, malfunctioning mitochondria, plaque deposition, and neuronal death. Amyloid plaque and other stimuli activate macrophages, which are crucial immune cells in AD development, triggering the production of inflammatory molecules and contributing to the disease's pathogenesis. The risk of AD is doubled by risk factors for atherosclerosis, dementia, advanced age, and type 2 diabetic mellitus (DM). As individuals age, the prevalence of neurological illnesses such as AD increases due to a decrease in glyoxalase levels and an increase in AGE accumulation. Insulin's role in proteostasis influences hallmarks of AD-like tau phosphorylation and amyloid β peptide clearance, affecting lipid metabolism, inflammation, vasoreactivity, and vascular function. The high-mobility group box 1 (HMGB1) protein, a key initiator and activator of a neuroinflammatory response, has been linked to the development of neurodegenerative diseases such as AD. The TLR4 inhibitor was found to improve memory and learning impairment and decrease Aβ build-up. Therapeutic research into anti-glycation agents, receptor for advanced glycation end products (RAGE) inhibitors, and AGE breakers offers hope for intervention strategies. Dietary and lifestyle modifications can also slow AD progression. Newer therapeutic approaches targeting AGE-related pathways are needed.

## Introduction and background

Alzheimer's disease (AD), a complex neurodegenerative condition, has been closely linked to the accumulation of advanced glycation end products (AGEs) in the brain. This association has sparked a review of novel therapeutic approaches aimed at mitigating AGE-induced neurodegeneration. Creating effective therapeutic strategies to combat this debilitating disease requires a comprehensive understanding of the complex interactions between AGEs and AD's pathology. AD accounts for 50-60% of the 35 million dementia cases reported around the world [1,2]. It is estimated in the study that 152 million individuals worldwide will have AD and other dementias by 2050. According to reports, Asian nations have reduced prevalence rates of AD [3]. The incidence rates are higher in females when compared to males; this is because of a decrease in antioxidant enzymes and post-menopausal hormonal changes [4,5].

In the cortical and medial temporal lobe areas of the brain, neurofibrillary tangles (NFT) and senile neuritic plaques are the typical indicators of this condition. N-terminally shortened β-amyloid, or the β-amyloid peptide, is what makes up extraneuronal senile plaques, whereas NFT is composed of hyperphosphorylated tau protein [6,7]. Another factor that leads to cell death in AD is the incapacity of these hyperphosphorylated proteins to bind to microtubules, which are in charge of maintaining the cell's structural integrity [8]. AD is frequently associated with hirano bodies, neutrophil threads, and cerebrovascular amyloid, as well as dystrophic neuritis, granulovacuolar degeneration, and loss of neurons and dendritic tissue [9]. Cholinergic neurostimulating drugs, β-amyloid precursor protein, and other eosinophilic, refractile substances combine to form Hirano bodies and aberrant microfilaments, β-amyloid precursor protein, hippocampus cholinergic neurostimulating peptide (HCNP), and TGFβ-3 [10].

There are various well-established neurodegenerative mechanisms contributing to the onset and course of AD, such as β-amyloid plaque deposition, abnormal translocation of protein tau, deregulation of Rho and p-21-activated kinases, activation of inflammatory mediators, and a decrease in acetylcholine levels [11,12]. In addition to the several pathogenic processes that lead to the onset of AD, extensive abnormalities in insulin and insulin-like growth factor (IGF) signaling apparently play a critical role in the development of AD and the functioning of the brain [13]. The most common causes of AD include increasing age, mutations in presenilin that may lead to decreased activity of gamma-secretase, and the generation of AGEs as a result of elevated blood glucose levels in the blood. Environmental factors, such as smoking, exposure to lead, head injury, insulin deficiency, and resistance in the brain, may also lead to AD [14-16]. The most common signs and symptoms of AD include progressive dementia, confusion, poor judgment, language disturbance, agitation, and hallucinations [17]. In 1975, Marshal Folstein et al. developed the mini-mental state examination (MMSE) in 1975 as a quick (5-10 min) tool for evaluating hospitalised patients' mental health. In the therapeutic context, it is regarded as the most popular test for standardised cognitive evaluation, particularly with the elderly population. AD can be categorised as mild, moderate, or severe based on the MMSE score [17].

## Review

AGEs and glycation: impact on chronic diseases and neurodegenerative disorders

AGEs represent a distinct class of oxidant substances that may lead to diabetes and other long-term diseases such as AD, cancer, atherosclerosis, and other inflammatory diseases. These are also called glycotoxins. Reduced sugars and free amino groups from proteins, fats, or nucleic acids react non-enzymatically to create AGEs. Glycation causes beta-sheet structures to develop in the hepatitis B surface [18]. There are six different types of AGEs that are classified based on the compound they originate from. AGEs formed from glucose can be termed AGE-1; those derived from glyceraldehydes are considered AGE-2; AGE-3 is derived from alpha dicarbonyls; AGE-4 is formed from methylglyoxal; AGE-5 is formed from glyoxal; and AGE-6 is formed from 3-deoxyglucosone [19]. AGEs are also seen in healthy individuals because they are a part of normal metabolism, but increased levels of AGEs in tissue may lead to diseases such as diabetes mellitus (DM), AD, and cancer [20]. Glycation results in an alteration of protein structure, which leads to the occurrence of major diseases such as diabetes and AD [21]. Different structures of AGEs, such as N-carboxymethyl lysine (CML), pentosidine, and pyrraline, may also lead to many degenerative processes or disorders such as AD and cataracts. The formation of AGEs may also lead to the generation of inflammatory mediators [22]. AGEs such as crossline were first observed in the kidney of a diabetic rat. These can be generated both within the body and in the laboratory by the reaction between glucose and free amino acids such as the epsilon amino group of lysine. AGEs such as CML have been predominantly present in neurodegenerative diseases [19]. Foods that consist of a high quantity of proteins and fats and foods cooked at high temperatures are the richest sources of AGEs [23]. AGEs formed from glucose can be termed AGE-1; those derived from glyceraldehydes are considered AGE-2; alpha-dicarbonyls are the source of AGE-3; methylglyoxal is the source of AGE-4; glycoxal is the source of AGE-5; and 3-deoxyglucosone is the source of AGE-6 [19]. AGEs such as crossline were first observed in the kidney of a diabetic rat. These can be generated both within the body and in the laboratory by the reaction between glucose and free amino acids such as the epsilon amino group of lysine. AGEs such as CML have been predominantly present in neurodegenerative diseases [19].

Receptors for advanced glycation end products (RAGE)

RAGE is a multiligand receptor that belongs to the immunoglobulin superfamily, encoded in the class III region of the major histocompatibility complex (MHC). Generally, these receptors have an extracellular region with C1, C2, and V domains and an intracellular region consisting of a cytoplasmic domain. Both of these regions are separated by a transmembrane layer. The V domain consists of two N-glycosylation sites that mostly bind to AGEs and other ligands, such as damage-associated molecular pattern (DAMP) molecules. Hence, this ligand is also called a pattern recognition receptor (PRR). Activation of the extracellular V domain leads to simultaneous stimulation of the cytoplasmic tail, which is responsible for activating intracellular signaling events [24], which eventually leads to the production of oxygen radicals that are highly reactive, inflammatory cytokines (IL-1, IL-6, and TNF-α), cell apoptosis, and further upregulation of RAGE. Thus, RAGE plays an important role in pathological conditions such as immune-associated diseases, including systemic lupus erythematosus, rheumatoid arthritis, pulmonary fibrosis, AD, diabetes, and cardiovascular disorders. Usually, RAGE distribution is found to be low in normal, healthy adults, but it seems to be elevated in individuals with certain pathological conditions such as cardiovascular diseases, cancer, and diabetes. Thus, recent therapeutic approaches aim specifically at inhibiting or blocking the extracellular-intracellular domains of RAGE [24,25].

**Figure 1:**
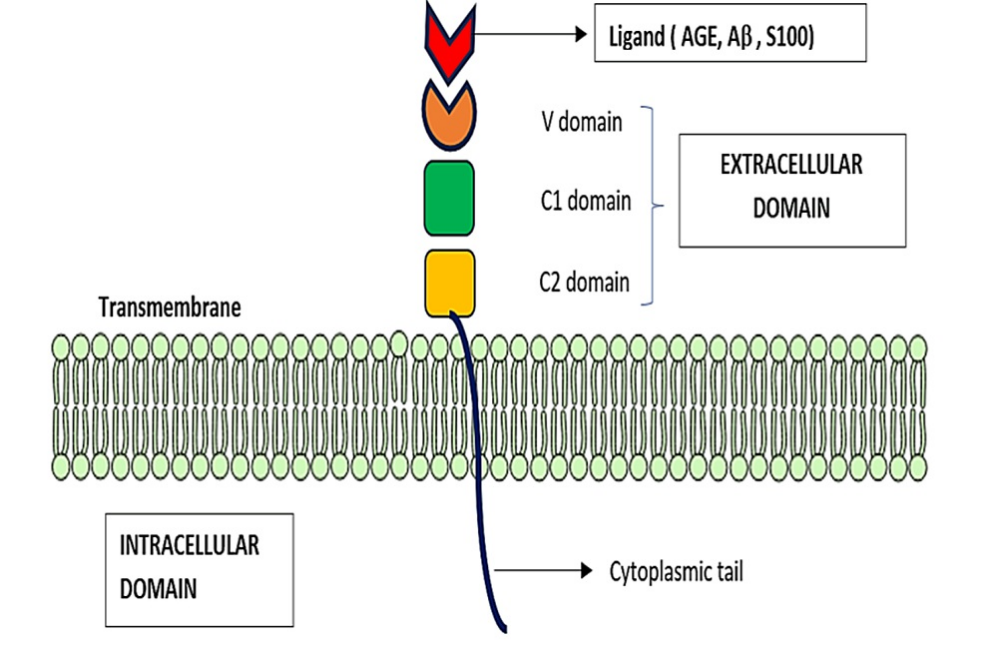
RAGE Structure RAGE - receptors for advanced glycation end products; AGE - advanced glycation end products; Aβ - amyloid beta

CML and carboxyethyl lysine (CEL) are primarily formed through the Maillard reaction. Among these substrates, carboxymethyl and carboxyethyl moieties act as AGEs that can bind to the positively charged V domain of RAGE in diabetes and AD sufferers [25]. The structure of RAGE is shown in Figure 1.

Toll-like receptor 4 (TLR4)

TLR4 is another PRR that is briefly involved in AD pathogenesis. The TLR4 signaling pathway is an important pathway for maintaining the inflammatory process and regulating the uptake and phagocytic elimination of Aβ plaques. Disruption of this pathway could be mainly because of a change in the microglia and macrophages' inflammatory state, which leads to the production of inflammatory cytokines (IL-1β, IL-6, and TNF-α) that damage the neurons in AD. Additionally, the presence of amyloid leads to the activation of the TLR4-mediated NF-κB/MAPK inflammatory axis, which results in the phagocytosis of amyloid by forming amyloid plaques [26].

RAGE ligands

AGEs

**Figure 2:**
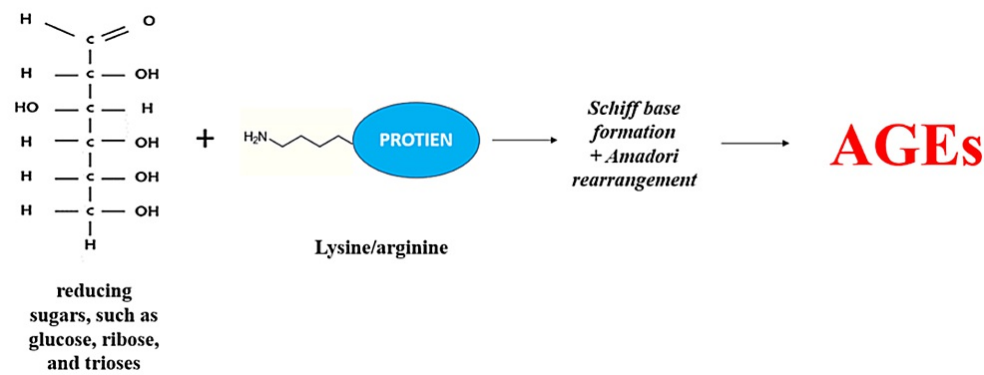
Nonenzymatic Reactions of Reducing Sugars With the Protein Amino Group AGEs - advanced glycation end products

**Figure 3:**
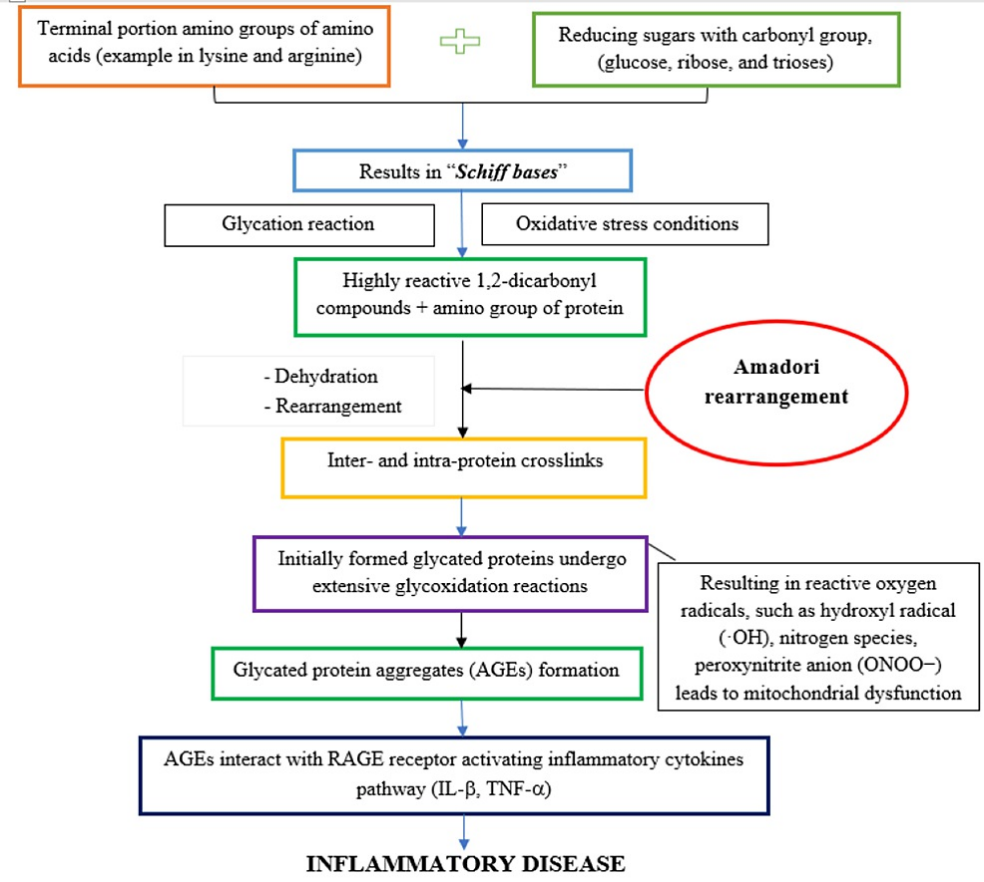
Protein Glycation Process – AGE Formation Through the Maillard Reaction AGEs - advanced glycation end products; IL - interleukin; TNF - tumor necrosis factor;

In a human body, glycation of proteins can occur in both normal and hyperglycaemic conditions and forms irreversible AGEs through a complex series of reactions involving in the formation of Schiff bases, the Amadori reaction, and the Maillard reaction. These steps eventually lead to the formation of toxic protein aggregates called AGEs [27], as shown in Figure 2. In the initial step, when proteins containing carbonyl groups from reducing sugars, such as glucose, ribose, and trioses, come into contact with the terminal amino groups of amino acids or the sidechain amino groups of lysine and arginine, a nonenzymatic reaction occurs that results in the formation of Schiff bases [25]. These Schiff bases then go through glycoxidation reactions under conditions of extreme oxidative stress, producing the extremely reactive dicarbonyl compounds, also known as Amadori rearrangement, which are stable and highly reversible ketoamines. The amino groups of the proteins react with these dicarbonyl molecules, creating intra- and interprotein crosslinks (through the process of dehydration and rearrangements). Maillard reactions are another name for the group of intricate events that lead to the development of AGEs. This results in the inactivation of the AGE-crosslinked proteins, including receptor proteins and enzymes, for regular physiological processes. AGEs are the term used to describe these protein changes as well as the small-molecule products that arise from their breakdown [27], as shown in Figure 3.

CML is thus far the most important AGEs that occurs in diabetes and AD sufferers, is the most important ligand for RAGE, and is a common biomarker for both diseases. CML and CEL are formed by the reaction between lysine and the terminal amino group, most commonly with glyoxal and methylglyoxal compounds, known as the Maillard reaction, respectively [25].

Most of the AGEs are noticed in NFT, senile plaques (5% AGEs), glial cells, and pick bodies [28-30]. Abnormalities in the brain glucose metabolism led to the formation of AGEs. The binding of these AGEs to the RAGE receptors may lead to the generation of proinflammatory cytokines, oxygen, and nitrogenous-based radicles, which is the main cause of high oxidative stress conditions. These cause mitochondrial dysfunction, deposition of plaques, and hyperphosphorylation of tau protein, causing neuronal death [20].

Glyceraldehyde-derived AGEs enhance the expression of amyloid precursor protein (APP) by the reactive oxygen species (ROS), which leads to neuronal death. Sirtuin 1 may protect the neuron by inhibiting ROS [31]. AGEs have the ability to block the proteasomal core, which leads to reduced proteolytic activity, and this is the main cause of protein accumulation in various parts of the body [28,32].

AGEs may lead to pathology through two mechanisms. First, AGEs cross-link proteins by changing the structure of proteins. AGEs are cleaved in the body by glyoxalase I and II through a process called enzyme degradation and eliminated by the kidney [33]. AGEs bind to the receptor for AGE (RAGE). Beta-amyloid protein can be transported across the blood-brain barrier (BBB) by RAGE, which leads to the development of AD [34]. Glycated proteins lead to the activation of microglia and macrophages; after that, they may lead to oxidative stress and the release of cytokines, which may cause the advancement of the disease [35].

**Figure 4:**
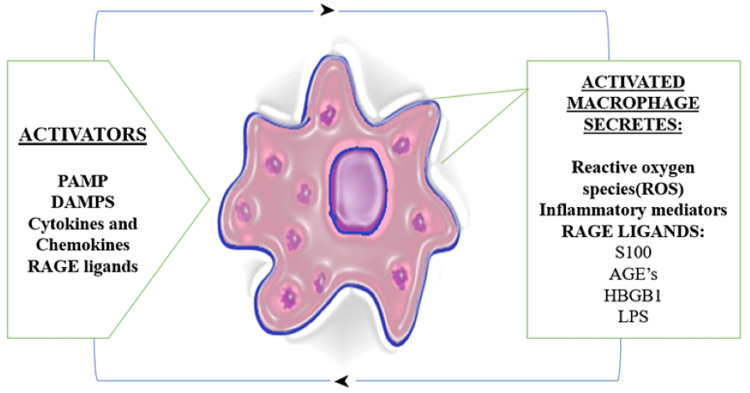
Activated Macrophages and AGE Production PAMP - pathogen-associated molecular pattern molecules; DAMPs - damage-associated molecular patterns; RAGE - receptor for advanced glycation end products; HMGB1 - high-mobility group box 1; LPS - lipopolysaccharides

Macrophage activation and AGE formation: Macrophage is an important immune cell that is involved in the progression of AD. The presence of the major component of amyloid plaque in AD and various other stimuli such as cytokines, ROS, and RAGE ligands such as AGEs, HMGB1, S100, and lipopolysaccharides (LPS) can activate a macrophage. This activated macrophage further produces inflammatory molecules, cytokines, and chemokines. Among cytokines, it includes the production of RAGE ligands, such as AGEs, HMGB1, S100, and Ab. Hence, the production of AGEs and its activation of macrophages in the body become a continuous vicious cycle in AD pathology [36], as shown in Figure 4.

HMGB1 and S100

High-mobility group box 1 (HMGB1) and S100 are RAGE ligands, also called DAMPs, secreted by activated macrophages and also by damaged and stressed cells. This can stimulate an immune reaction and is perceived by the DAMP receptor, including RAGE receptors. HMGB1 has been shown to interact with Aβ peptides, promoting their aggregation and enhancing their neurotoxicity. Additionally, HMGB1 can stimulate the release of Aβ from microglial cells, further contributing to Aβ accumulation. HMGB1 has been implicated in BBB dysfunction. BBB allows the entry of peripheral immune cells and inflammatory molecules to reach the brain if the barrier is disrupted, aggravates neuroinflammation, and potentially contributes to AD pathogenesis [26,36].

S100 proteins are calcium-binding proteins. Among this family, one specific member, S100B, has been involved in AD pathology. S100B can activate the immune cells of the brain called astrocytes and microglia, leading to neuroinflammatory conditions. Activated glial cells will release pro-inflammatory cytokines, such as interleukin-1 beta (IL-1β) and tumor necrosis factor-alpha (TNF-α), one of the main contributing factors to inflammatory response that is seen in AD. Higher levels of S100B are directly related to neurotoxic effects, and this can induce neuronal apoptosis and production of ROS, causing oxidative stress and damage to neurons [24,26].

Risk factors of AD

The various risk factors that lead to AD are increased blood pressure and hypercholesteremia in adults. These risk factors may induce atherosclerosis, which leads to decreased blood flow to the brain, which is the major cause of dementia. Using anti-inflammatory drugs may also be a risk factor for AD [37]. Increased age and type 2 DM increase the risk of AD by two-fold. Mutation in the APP gene located on chromosome 21 and the presence of apo lipoprotein E4 are also risk factors for AD [38,39]. Increased plasma homocysteine levels due to low vitamin B levels also put individuals at higher risk of developing dementia [40]. Other risk factors include stroke, body weight fluctuations between low and high, smoking, limited physical activity, AGE-forming food consumption, high-calorie food consumption, poor dietary folate intake, sedentary lifestyle, and low estrogen levels [41,42].

Mechanisms of risk factors common for AD and AGEs

DM

DM can cause inflammation and insulin resistance, which raise blood glucose levels and cause non-enzymatic glycation, protein and lipid oxidation, and the creation and buildup of AGEs. The interaction between these produced AGEs and RAGE can lead to the production of ROS, which in turn causes oxidative stress, the primary cause of cell death that ultimately results in dementia [43-45].

Age

AGEs have been linked to a number of diseases, such as aging and inflammatory skin conditions. They are created by substances such as methylglyoxal, glyoxal, and ultraviolet B (UVB). Alzheimer's and other neurological illnesses are more common as people age because of a drop in glyoxalase levels and an increase in AGE accumulation [46]. In older persons, both with and without diabetes, higher peripheral AGE levels are linked to a higher rate of cognitive deterioration. In older adults, methylglyoxal, the precursor, also has a major impact on neurodegeneration and cognitive loss. Extracellular proteins and cytoskeletal proteins, which are in charge of preserving cellular integrity, are the primary targets of AGEs. The primary cause of AD, cell death, may result from a reduction in the binding of cytoskeletal proteins to microtubules caused by the loss of these proteins [47,48].

Smoking

According to a study, tobacco's aqueous extract contains ROS and has the most affinity for lipids and proteins. Glycated and oxidative chemicals are produced by the Maillard reaction cascade when the tobacco leaves are dried in the presence of sugars. These produced AGEs evaporate during combustion and are inhaled by the lungs as smoke, where they react with serum protein [49]. As previously mentioned, smoking also causes more AGEs to be formed. These AGEs are mostly deposited in the skin, where they speed up the aging process and cause cell death [50,51].

Diet

Foods heavy in protein (such as red meat, cheese, and chicken), high in carbohydrates, and foods cooked at high temperatures (such as frying and broiling) have a high propensity to generate advanced glycation end products. Cooking may cause more AGEs to develop because of the body's interaction between amino and carbonyl groups, which causes cell death [18,52].

Atherosclerosis 

Atherosclerosis advances as a result of AGEs. Plaque deposition is the primary cause of the formation of atherosclerotic lesions. When AGEs build up in an atherosclerotic lesion, they induce endothelial dysfunction by way of the pro-apoptotic impact [49,53]. In addition to increasing inflammatory mediators' adhesion to the vessel wall and p50 accumulation, which causes cell death, AGEs also upregulate the expression of monocyte chemoattractant protein-1, intercellular adhesion molecule 1, vascular cell adhesion molecule 1, and plasminogen activator inhibitor 1 [54,55].

Progressive novel therapeutic approaches against AD

HMGB1 Neutralization in AD

There are three main novel therapeutic mechanisms that have been identified to inhibit the production of HMGB1 produced extracellularly by activated macrophages, which may trigger various inflammatory receptors, particularly RAGE receptors, in AD [26]. The blockade or inhibition of HMGB1 is done by anti-HMGB1 monoclonal antibodies (mAb), specific HMGB1 inhibitors, and HMGB1 interference (shRNA). Anti-HMGB1 mAb therapy has been particularly beneficial in animal models with brain inflammation and BBB disruption [36]. It helps in the reduction of Ab aggregates and in its phagocytosis. HMGB1, such as glycyrrhizin and its derivatives, has been shown to reduce the inflammatory markers (TNF-α and IL-1β mRNA) and protein expression of COX-2 and iNOS. HMGB1 short hairpin RNA (shRNA) is involved in the inhibition of the translocation process of HMGB1 from the nuclei to the cytoplasmic membrane [26].

RAGE Inhibition in AD

Azeliragon (TTP488) or PF-04494700 is a RAGE inhibitor, and its chemical name is 3-[4-[2-butyl-1-[4-(4-chlorophenoxy) phenyl] imidazol-4-yl] phenoxy. TTP488 is an orally bioavailable small molecule that is still under investigation and prevents the binding of RAGE ligands such as Ab, AGEs, S100, and HMGB1 to the RAGE receptor, thereby inhibiting the activation of the inflammatory signal. Along with this action, TTP488 was also found to inhibit neurocognitive function and neuronal Ab accumulation [26]. However, in 2007, a large 18-month phase II trial included 399 subjects who had mild-to-moderate AD to evaluate the effectiveness of TTP488. This trial showed a decline in cognitive response compared to the lower dose group, which showed a mild, progressive decline. Hence, this study was discontinued at the end of six months [56-58].

In a study, they came up with a sequence of dual RAGE/SERT inhibitors as a promising treatment for the AD group with depression as a comorbidity. TTP488 is the first among RAGE inhibitors to enter clinical phase Ⅲ as a disease-modifying drug against AD, and vilazodone is a SERT inhibitor that is approved as an antidepressant for the treatment of major depression. They showed about 12 optimal compounds with good safety and efficacy through multiple assays and concluded that these compounds could be a prospective prototype for AD and depression comorbidity [59-62].

FPS-ZM1 (4-chloro-N-cyclohexyl-N-(phenylmethyl)benzamide) is another RAGE inhibitor. This small molecule can bind to the RAGE receptor; as a result, production of Aβ40 and Aβ42 can be inhibited. Simultaneously, it inhibits β-secretase activity, an enzyme that is involved in the formation of Aβ peptides, therefore preventing brain damage [26].

TLR4 Blockade in AD

TLR4 activation can lead to neuroinflammation in AD. TLR4 blockade or inhibition, done by molecules by inhibiting TLR4 expression, preventing microglial activation, and depressing the levels of pro-inflammatory cytokines, improving learning and memory functions, has been noticed as a result of inhibiting oxidative stress, therefore reducing apoptotic cell death and Aβ load in the brain. There are ongoing pre-clinical studies investigating various molecules for the blockade of TLR4 in AD that have not yet advanced to clinical trials involving human subjects [63,64].

Insulin

One of the essential features of type 3 diabetes mellitus is insulin resistance; therefore, insulin sensitivity-improving strategies will be beneficial for early-stage AD patients who are at risk of developing the disease. Peripheral insulin administration, which is used for treating diabetes, may cause hypoglycaemia and might also be ineffective due to its limited BBB insulin transport. Thus, recent investigation has shifted their focus to intranasal insulin administration; here, the insulin is delivered through trigeminal perivascular and olfactory nerve channels to the brain, evading the BBB. In rodent models, intranasal insulin administration has improved the AD pathology and retained both long-term and short-term memory [65-67]. Insulin sensitivity improving strategies to treat AD are exhibited in Table 1.

**Table 1:** Insulin Sensitivity Improving Therapeutic Strategy for AD AD - Alzheimer's disease; CNS - central nervous system; APOE - apolipoprotein E; APP - β-amyloid precursor protein

S. No	Drug	Study design	Results	Authors
1.	Intranasal insulin	Double-blind placebo-controlled design (study started with device 1 and switched to device 2).	The neuroimaging results for the device 2 insulin arm demonstrated minor but significant reductions in hippocampal volume and corresponding reductions in entorhinal cortex volume.	Craft et al., 2020 [65]
2.	Intranasal insulin	Preliminary single-site trial	The study suggests that intranasal insulin can enhance memory and cognitive function in AD, but further research is needed to explore long-term effects and its applicability to patients with more advanced disease. The study also observed changes in plasma insulin and amyloid-beta (Aβ) levels, indicating potential interactions between CNS insulin signaling and peripheral insulin regulation.	Reger et al., 2008 [66]
3.	Insulin (intravenous injection)	Cross-sectional study	The study observed that normal individuals and those with the ε4 allele of the APOE gene showed a decrease in APP when insulin levels were raised to about 35 µU/ml, a concentration similar to what is achieved after a meal. However, AD patients without the ε4 allele only showed a decrease in APP when insulin levels were raised to 85 µU/ml, a higher level that may be observed after high caloric intake or in the context of insulin resistance.	Craft et al., 2003 [67]

Glucagon-Like Peptide-1 Receptor (GLP-1R) Agonists

GLP-1R agonists, commonly used in managing diabetes, have shown neuroprotective effects in preclinical and early clinical studies. These agents have demonstrated the potential to enhance neuronal survival, reduce neuroinflammation, and improve cognitive function. Further investigations are needed to determine their disease-modifying potential and optimize their use in neurodegenerative diseases.

Exendin-4/exenatide: Preclinical and early clinical research on GLP-1R agonists, which are frequently used to treat diabetes, has revealed neuroprotective properties. There is evidence that these medicines can decrease neuroinflammation, increase neuronal survival, and boost cognitive performance. Short-acting GLP-1 Ras, Ex-4, promotes brain insulin and insulin signaling in type 2 diabetes and decreases AD-like tau hyperphosphorylation. Synthetic versions of exendin-4, called exenatide and exendin-4, have been shown to alleviate cognitive impairment in AD models, guard against inflammation, boost cholinergic function, and preserve hippocampal neurons. In differentiated human neural progenitor cells, exendin-4 can also traverse the BBB and shield neurons against AβO-induced death. Treatment with extendin-4 may improve memory and cognition in rats and prevent tau hyperphosphorylation associated with AD via controlling the insulin signaling system [68].

Liraglutide (LRGT): LRGT, an analog of GLP-1, has been thoroughly investigated in AD animal models. The peripheral and central nervous systems are engaged in the regulation of glucose levels and metabolic balance by GLP-1. GLP-1 receptor agonists are examples of insulinotropic hormones that promote insulin secretion and control glucose levels. GLP-1 receptor agonists are found in the brain, in which they regulate GLP-1 functions that protect against excitotoxicity and apoptosis while facilitating cell growth and proliferation. Type 2 diabetes patients often utilize LRGT to decrease glucagon and enhance β cell activity, which restores normoglycemia. It is noted that LRGT possesses anti-inflammatory effects and can balance out changes in brain metabolism [64].

Currently, a phase IIb study, called evaluating LRGT in AD trial, is being conducted on patients with mild AD. It is associated with improvements in MRI in specific brain volumes and tau-P181/Aβ42 ratio. Promising results, including the preservation of general cognition, have also been reported following intranasal insulin injection [68,69]. The other available therapies to treat AD against AGEs are presented in Table 2.

**Table 2:** Other Available Therapies Against AGEs in AD iNOS - inducible nitric oxide synthase; AGE - advanced glycation end products; NO - nitric oxide; PGE2 - prostaglandin E2; COX-2 - cyclooxygenase 2; NF-κB - nuclear factor kappa-light-chain-enhancer of activated B cells; DNA - deoxyribonucleic acid;OPB-9195-(+/-) - 2-isopropylidenehydrazono-4-oxo-thiazolidin-5-ylacetanilide; RAGE - receptor for advanced glycation end products

Drug	Category	Mechanism of action	Author
AMINOGUANIDINE (AG)	Inducible nitric oxide synthase (iNOS) inhibitor	Aminoguanidine decreased the progression of Alzheimer disease induced by β-amyloid; AGE decreases the production of NO, PGE2, COX-2, and NF-κB.	Ooi et al., 2013 [30]
PYRIDOXAMINE (PM)	Quenching of protein dicarbonyls	By trapping reactive oxygen species (ROS), PM inhibits the oxidation of proteins and DNA directly and the production of AGEs from Amadori adducts.	Herrero et al., 2011 [58], Chiazza et al., 2017 [61]
BENFOTIAMINE	Lipid soluble analogue of thiamine	Benfotiamine inhibits three key pathways involved in vascular damage due to hyperglycemia. Benfotiamine activates an enzyme called transketolase, which promotes the shunting of AGE precursors toward the pentose phosphate pathway, therefore lowering AGE formation.	Gibson et al., 2020 [62]
OPB-9195	Thiazolidine derivative that supresses the AGEs formation.	In reactive carbonyl compounds their carbonyl groups nonenzymatically react with protein amino groups to form AGEs, OPB-9195 to prevent this forms carbonyl intermediates thereby reducing the production of AGEs.	Miyata et al., 2000 [63]
ALGAEBRIUM	Reverse the protein crosslinks in AGEs	Algaebrium breaks methylglycoxal mediated AGE protein crosslinks.	Reddy et al., 2022 [25]
CURCUMIN	Polyphenol	It suppresses the expression of the RAGE gene, resulting in less display of AGEs involvement in oxidative stress in cells and lowering AGEs inflammatory response by inhibiting the activation of nuclear factor-kB.	Alizadeh et al., 2017 [64]

## Conclusions

A crucial contributing factor to the onset and development of AD is AGEs. The need for focused treatment strategies is emphasised by the harmful consequences of AGEs on the brain, which include oxidative stress, inflammation, the development of amyloid-beta plaques, and the creation of neurofibrillary tangles. Ongoing therapeutic research into anti-glycation agents, RAGE inhibitors, and AGE breakers provides hope for intervention strategies that can significantly reduce the impact of AGE accumulation in the brain. Furthermore, dietary and lifestyle modifications can lower AGE production, and accumulation offers a promising avenue for preventing or slowing the progression of AD. A concentrated effort must be made to investigate newer therapeutic approaches that especially target AGE-related pathways, given the complex role that AGEs play in the pathogenesis of AD.
